# A cell-free approach to identify binding hotspots in plant immune receptors

**DOI:** 10.1038/s41598-021-04259-8

**Published:** 2022-01-11

**Authors:** George C. Markou, Casim A. Sarkar

**Affiliations:** 1 Department of Chemical Engineering and Materials Science, University of Minnesota, Minneapolis, MN 55455 USA; 2 Department of Biomedical Engineering, University of Minnesota, Minneapolis, MN 55455 USA

**Keywords:** Plant immunity, Molecular engineering

## Abstract

Plant immune receptors are often difficult to express heterologously, hindering study of direct interactions between these receptors and their targets with traditional biochemical approaches. The cell-free method ribosome display (RD) enables expression of such recalcitrant proteins by keeping each nascent polypeptide chain tethered to its ribosome, which can enhance protein folding by virtue of its size and solubility. Moreover, in contrast to an in planta readout of receptor activity such as a hypersensitive response that conflates binding and signaling, RD enables direct probing of the interaction between plant immune receptors and their targets. Here, we demonstrate the utility of this approach using tomato recognition of *Trichoderma viride* ethylene-inducing xylanase (EIX) as a case study. Leveraging the modular nature of the tomato LeEIX2 and LeEIX1 leucine-rich repeat (LRR) receptors, we applied an entropy-informed algorithm to maximize the information content in our receptor segmentation RD experiments to identify segments implicated in EIX binding. Unexpectedly, two distinct EIX-binding hotspots were discovered on LeEIX2 and both hotspots are shared with decoy LeEIX1, suggesting that their contrasting receptor functions are not due to differential modes of ligand binding. Given that most plant immune receptors are thought to engage targets via their LRR sequences, this approach should be of broad utility in rapidly identifying their binding hotspots.

## Introduction

The plant immune system is innate but highly diverse and dynamic in its protective role. In the plasma membrane and cytoplasm of each cell, dozens to hundreds of unique receptors scan their milieus to identify potential threats. Most of these plant immune receptors are leucine-rich repeat (LRR)-containing proteins^1^, including the family of transmembrane LRR receptor-like proteins (RLP). RLPs effect phenotypic changes in the plant in response to molecular sensing, often in cooperation with LRR receptor-like kinases as RLPs themselves lack a kinase domain^2^. The repetitive 20–30 amino acid LRR motifs of RLPs stack together to form modularly-composed extensive solvent-exposed surfaces, ideal for binding target proteins and other biomolecules. Interactions along these surfaces are key to our understanding of plant immunity^3^.

**Figure 1:**
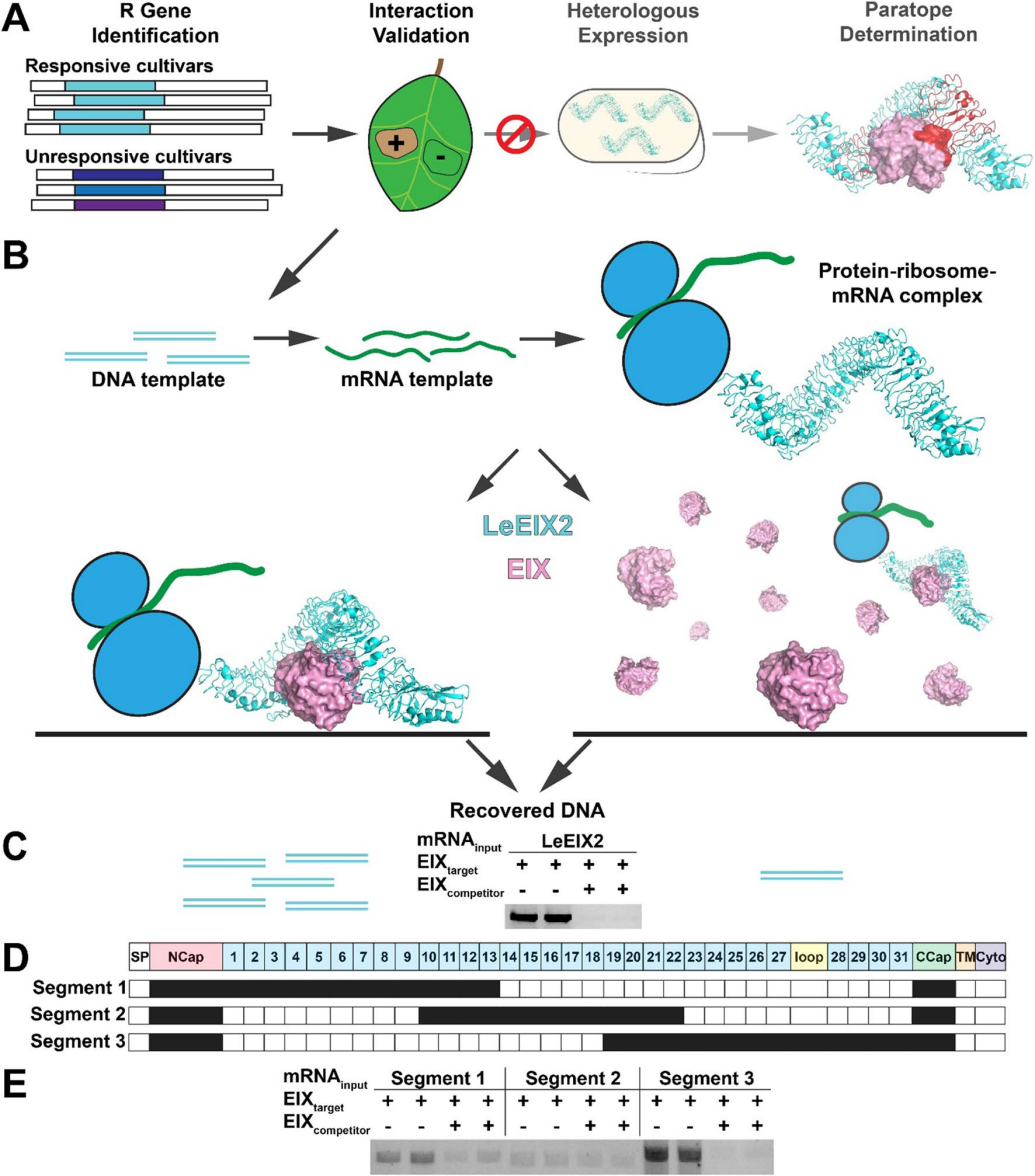
Flowchart for LRR paratope mapping. (**A**) The existing workflow for study of LRR immune protein-target binding involves identification of the R gene of interest and phenotypic interaction validation. The immune receptor is then heterologously expressed for further biochemical analysis. This expression often fails, and the workflow ceases with no path forward. (**B**) RD enables more detailed biochemical study of recalcitrant immune receptors by providing an alternative, cell-free route for interaction analysis. The gene, *LeEIX2* in this case, was amplified, transcribed, and translated to create a protein-ribosome-mRNA heterotrimeric complex. This ribosomal complex was panned against immobilized target EIX either in the absence or presence of excess EIX in solution acting as a competitor. Upon washing, unbound complexes were removed from the wells. (**C**) Bound complexes were reverse transcribed and PCR amplified; products were visualized via agarose gel electrophoresis for RD technical duplicates. (**D**) LeEIX2 ectodomain partitioning into three segments, each defined by the N-cap, 13 continuous LRRs, and the C-cap. (**E**) RD results of LeEIX2 segment binding of immobilized EIX in the absence or presence of excess competitor EIX. Agarose gel electrophoresis results are shown for RD technical duplicates.

Despite their importance, there has been limited success in identifying LRR regions involved in target recognition, the receptor paratope(s). In vivo mutational analysis is convoluted by mutant stability and coupled binding-signaling phenotypic readout^4,5^. The gold standard of X-ray crystallography has yielded few structures with cognate ligands, as heterologous protein overexpression and purification has been unsuccessful in most cases, barring a few select receptors expressed in insect cell lines^6–9^. Thus, there is a need for an alternative approach to study the biochemical determinants of target recognition in recalcitrant plant immune receptors (Fig. 1A).

Ribosome display (RD) is a powerful protein engineering platform that is particularly well suited to analyzing and engineering proteins that are difficult to express and properly fold^10^. The method entails cell-free expression of a protein of interest without release from its translating ribosome, resulting in a stabilized complex of the protein with its ribosome and cognate mRNA. The ribosomally-displayed protein can be tested for function (e.g., target binding), and the coupled genetic material can be amplified and/or quantified to yield a measure of the protein’s function. Notably, since the ribosome itself is large and highly soluble, a protein that remains physically tethered to it has enhanced solubility and folding, including a reduced propensity for aggregation. This has been shown for amyloid-like proteins and even for a mammalian LRR protein^11,12^.

We reasoned that RD could offer a viable route to overcome heterologous expression challenges associated with plant LRR receptors and facilitate the study of their ligand-binding properties. LeEIX2, a 31-unit LRR-RLP from tomato that recognizes *Trichoderma viride* ethylene-inducing xylanase (EIX) in the apoplast, was chosen as a case study for this method. Its direct binding to EIX target in the absence of native protein context and lack of a solved crystal structure motivated this choice^13^. Using RD, we unexpectedly identified two distinct EIX-binding hotspots in LeEIX2 and in the decoy receptor LeEIX1.

## Results

### The LeEIX2 receptor-EIX effector interaction can be reproduced in ribosome display

In order to study the LeEIX2-EIX interaction, this direct binding event had to be reproduced in a cell-free context. We hypothesized that the LeEIX2 ectodomain should be sufficient, as it excluded the signal peptide, transmembrane, and cytosolic domains, which are unlikely to bind EIX but may adversely impact functional protein expression on the ribosome. Notably, the displayed amino acid sequence includes the N-terminal and C-terminal “caps” of the LeEIX2 ectodomain, which bury the hydrophobic cores of the first and last LRRs, mitigating the risk that unnaturally exposed hydrophobic residues may hinder proper folding or drive nonspecific interactions in binding assays. Each of the LeEIX2 protein domains and LRR units were defined in accordance with the study that first identified the receptor^13^.

The LeEIX2 ectodomain was expressed in RD format, and the resulting ribosomal complexes were panned against immobilized EIX either without or with excess EIX at a concentration of 1.25 µM in solution to produce total and nonspecific binding in the assay, respectively (Fig. 1B). After washing to remove unbound complexes, RT-PCR and subsequent gel electrophoresis of the remaining bound complexes provided a measure of mRNA retained due to binding of the LeEIX2 complex to EIX. A distinct signal difference between uncompeted and competed wells indicated specific binding to EIX (Fig. 1C, Supplementary Fig. 1). As additional validation, the LeEIX2 ribosomal complexes were separately panned against an immobilized unrelated target protein, maltose-binding protein (MBP), which did not generate a significant signal (Supplementary Figs. 2 and 3).

Notably, the full LeEIX2 ectodomain (31 LRRs in addition to the terminal caps and loopout domain) is ~ 115 kDa, which to our knowledge is the largest protein functionally displayed on the ribosome. This required numerous technical advances to overcome the inherently poor signal-to-noise ratio in RD for large proteins^14^. First, we used the reconstituted PURExpress cell-free translation system (New England Biolabs) to enhance the signal since it is a minimal expression system that, in contrast to cell lysates, significantly reduces undesired components such as nucleases and proteases. Second, codon optimization of the LeEIX2 template also boosted the signal by improving the efficiency of translation in the *E. coli*-based system^15^. Third, the use of a protein-free blocking buffer, SynBlock (Bio-Rad), reduced nonspecific interactions that contribute to the noise compared to less inert but commonly used blocking agents such as bovine serum albumin or casein. Finally, Mfold was used to optimize the mRNA sequence for the reverse transcription step to maximize product yield^16^ (Supplementary Fig. 4A,B). These collective advances facilitated clear detection of the full ectodomain of LeEIX2 on the ribosome.

### LeEIX2 segmentation reveals two disjoint EIX-interacting hotspots

Having recreated the full LeEIX2 ectodomain interaction with EIX using RD, we sought to identify the subset of LeEIX2 LRRs that drive binding. Given the putative globular fold of EIX, our initial hypothesis was that the binding paratope in LeEIX2 would lie within a contiguous set of LRRs, so we employed a pseudo-branch and bound algorithm that leveraged the modularity of the repeat unit architecture. The 31-LRR LeEIX2 was split into three 13-LRR segments with partial overlap (Fig. 1D), and each segment was assayed in RD for the signal difference between uncompeted and competed wells to identify specific binding. Each segment shared 4 LRRs with its neighboring segment(s) to lessen the impact of potential misfolding of the outermost LRRs due to imperfect repeat stacking with the LeEIX2 N- and C-terminal caps, which were appended to all constructs. These initial RD experiments showed that both the first (LRRs 1–13) and the third (LRRs 19–31) segments exhibited specific binding to EIX (Fig. 1E, Supplementary Fig. 1).

### Model-informed design of receptor sub-segmentation

To refine the location of these two paratopes within LeEIX2, we sought to further partition the first and third LRR segments for additional RD experimentation. Given that there are numerous ways to further subdivide these segments for experimental testing, we sought to develop a strategy that maximized the information gained from these experiments while maintaining the binding properties of the repeat units.

In subdividing segment 1, we focused on the 11-LRR region of LRRs 1–11, since LRRs 12 and 13 were considered unlikely interactors based on the non-interaction of the second LeEIX2 segment with EIX. Likewise, in subdividing segment 3, we focused on the region from LRRs 21–31, as LRRs 19 and 20 were unlikely EIX interactors. To account for the additional loopout domain in segment 3, we treated it as a 12-LRR region that needed to be subdivided.

Initially, a biology-agnostic approach was employed to determine how much information could be obtained from an experiment in which a segment of set length *L* (number of repeats) was partitioned into sub-segments of length *n* (number of consecutive repeats). The experiment would thus consist of *L – n* + 1 unique sub-segments of equal length. Each individual repeat unit in the segment, *i*, would be included in some tested constructs and excluded in others, with *P*_*i*_ describing the fraction of constructs that would include repeat *i* in EIX binding assays. The information entropy for each possible experimental setup was calculated as

$$entropy= -\sum_{i=1}^{L}{P}_{i}\,{\mathrm{log}}_{2}\,{P}_{i}$$

**Figure 2:**
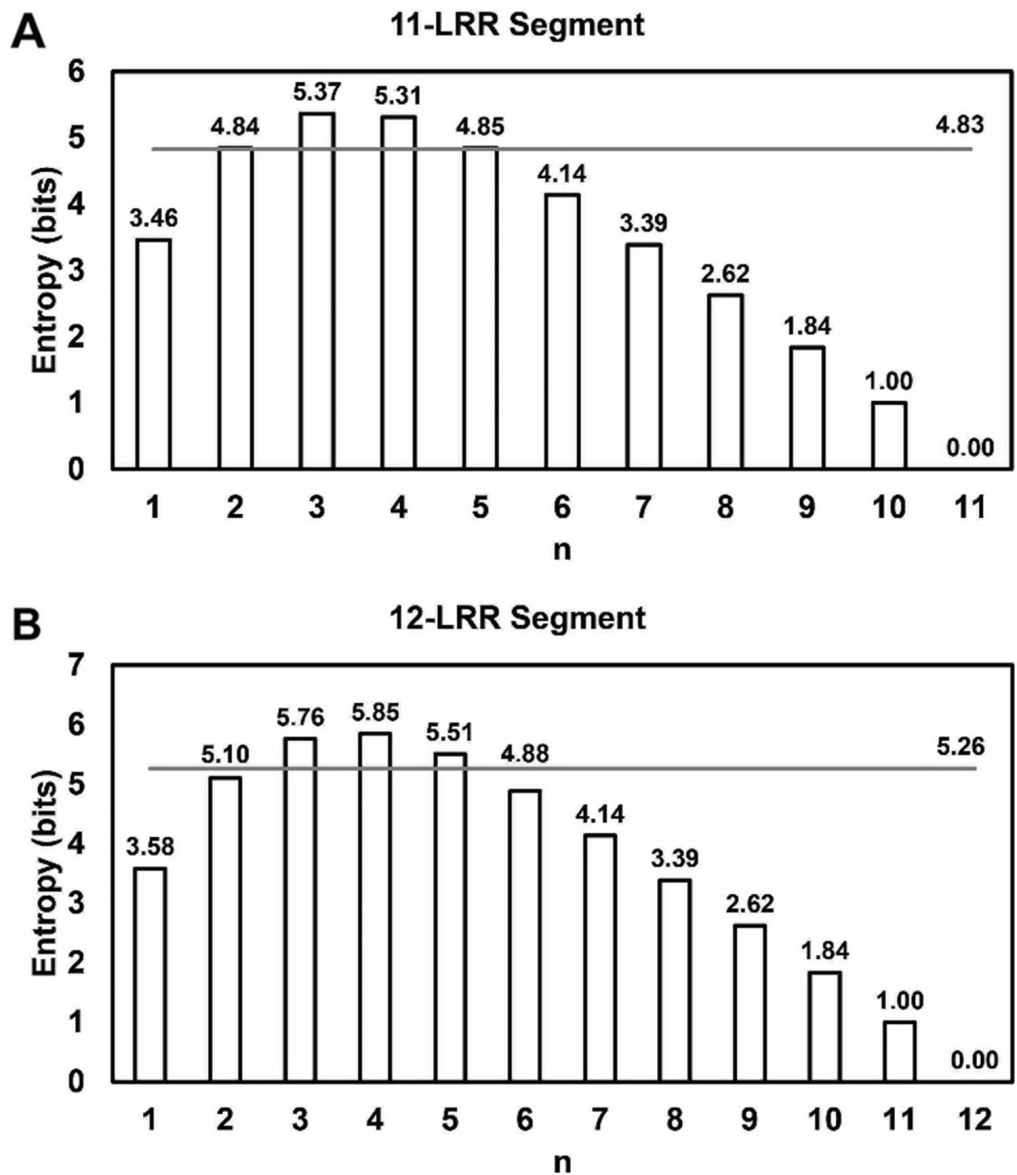
Information entropy for different LRR segmentation lengths. Information entropy for experimentation involving sub-segments of length *n* (number of repeats) from a segment of length (**A**) 11 or (**B**) 12. The grey bar indicates a level above which at least 90% of maximal information is obtained.

This calculation was performed for both 11- and 12-LRR segments (Fig. 2A,B). For LeEIX2 segment 1, maximal entropy occurs at *n* = 3, whereas the entropy for segment 3 is maximized when *n* = 4. We set an entropy tolerance of 90% of the maximal entropy, such that sub-segments with *n* = 2, 3, 4, 5 were considered for segment 1 and sub-segments with *n* = 3, 4, 5 were considered for segment 3. This algorithm is generalizable to inform unbiased segmentation of a repeat protein of any length.

Bringing in the biological context, we expected that non-native repeat unit-cap interfaces would be imperfect matches. Accordingly, repeats further from a mismatched interface are more likely to have native biological structure and function. There was also a concern that using too small of an *n* might reduce the interfacial contact area so much as to prevent detectable binding. Therefore, by testing sub-segments of *n* = 5, we were able to obtain near-maximal information while allowing for a more true-to-native biological environment for the repeats in each sub-segment.

### Modular sub-segmentation refines the interacting repeat units

**Figure 3:**
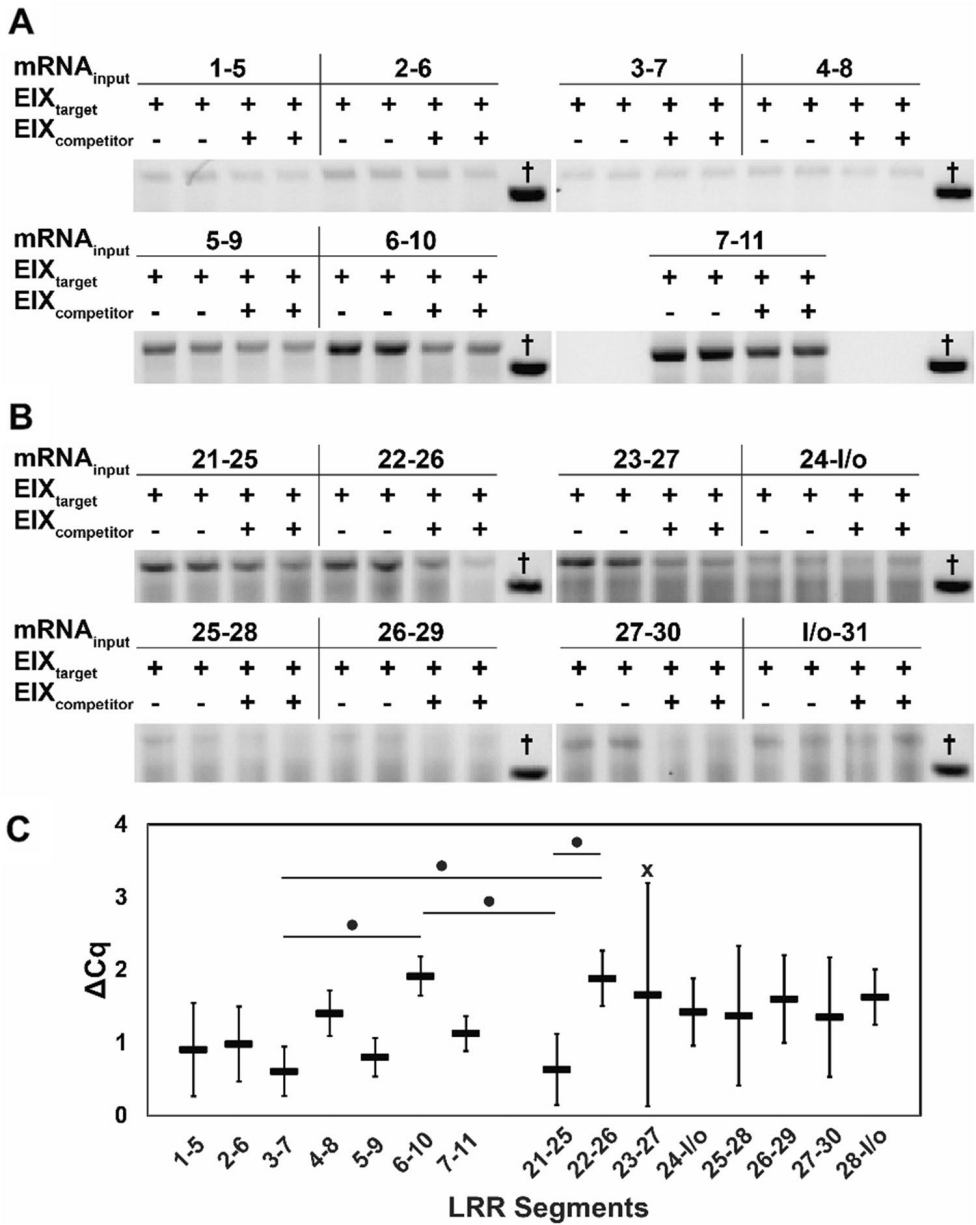
5-LRR sub-segment mapping. Gel electrophoresis results for RD technical duplicates of (**A**) LRRs 1–5 to 7–11 or (**B**) LRRs 21–25 to loopout (l/o)-31 panning against immobilized EIX target in the absence or presence of excess competitor EIX. An unrelated RT-PCR sample (†) serves as a loading control so that signal intensities across gels can be compared. (**C**) ΔCq means calculated from normalized qRT-PCR replicates measured in 5-LRR sub-segment RD experiments. Error bars represent replicate standard deviations. ^•^p ≤ 0.1 for Tukey’s method pairwise comparison of ΔCq following single-factor ANOVA. A high-variance data point (x) was identified as an outlier by Tukey’s heuristic and excluded from statistical analysis.

RD of these 5-LRR sub-segments against EIX confirmed the presence of two disjoint EIX-binding hotspots, with LRRs 6–10 and LRRs 22–26 showing the greatest signal-to-noise differences (Fig. 3A,B, Supplementary Fig. 5 and 6). These results were further validated by qRT-PCR, from which a mean ΔCq was calculated as the Cq of the competed wells minus the Cq of the uncompeted wells for each mRNA input tested (Fig. 3C, Supplementary Fig. 7). Again, LRRs 6–10 and 22–26 were found to have statistically significant signal differences between competed and uncompeted wells. The strong signals and high signal-to-noise ratios provide evidence for these two EIX-interacting sites. Although RD has been effectively used to express a number of functional proteins that are otherwise intractable^11,12^, low signals are more difficult to interpret, as there is no clear method to differentiate lack of function from protein misfolding on the ribosome.

**Figure 4:**
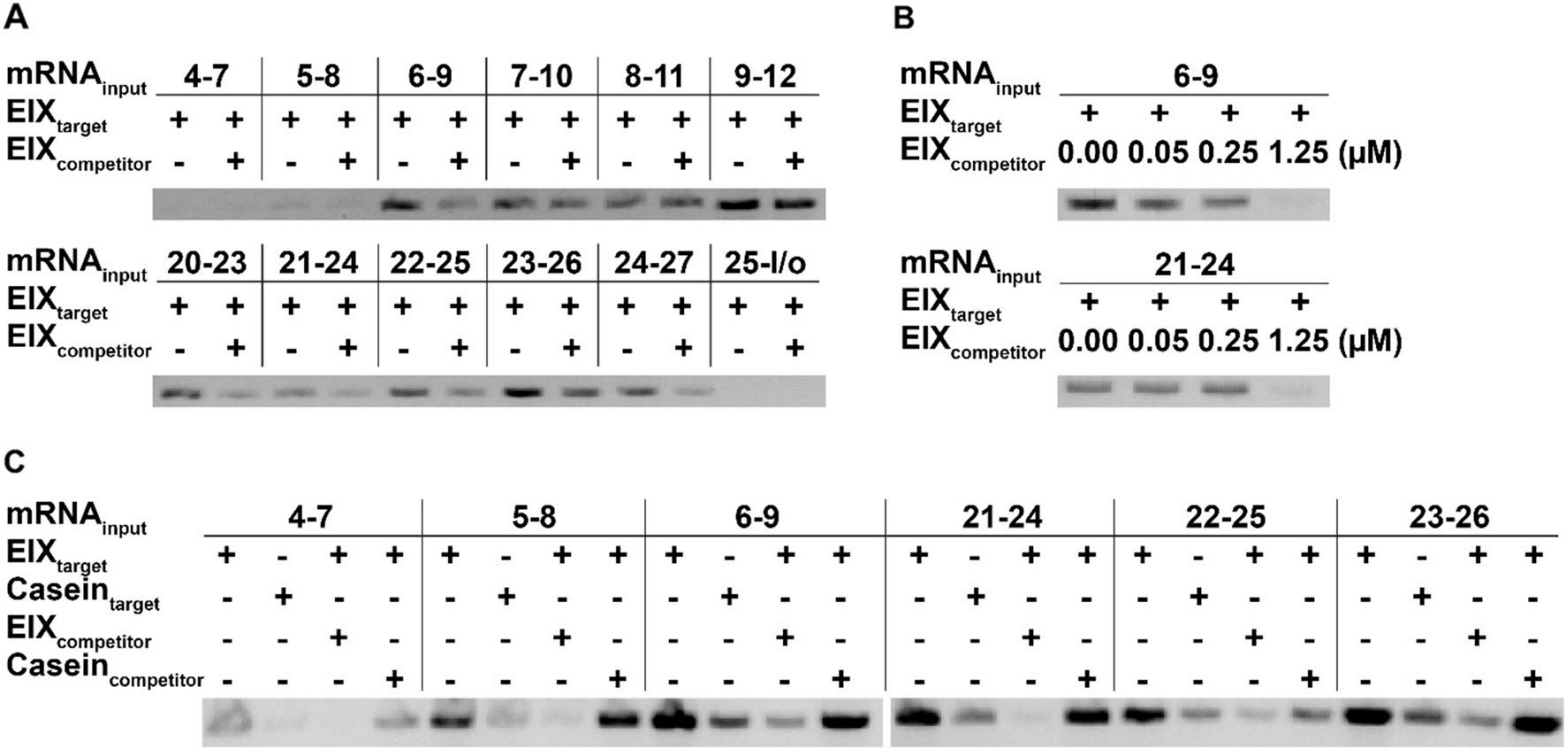
4-LRR sub-segment mapping. (**A**) RD results of 4-LRR LeEIX2 sub-segment/EIX binding signal in the absence or presence of competitor EIX in solution (‘l/o’ refers to loopout domain). (**B**) RD results of dose-dependent LeEIX2 sub-segment/EIX binding signal for sub-segments 6–9 (top) and 21–24 (bottom). Lanes are for varying EIX competitor concentration in solution. (**C**) RD results of 4-LRR LeEIX2 sub-segment binding signal against immobilized EIX, immobilized unrelated target casein, immobilized EIX in the presence of excess EIX competitor, and immobilized EIX in the presence of excess casein competitor.

Further experimentation for hotspot refinement was conducted by branching of neighboring 4-LRR segments (Fig. 4A, Supplementary Fig. 8), with these results generally in agreement with those from 5-LRR sub-segments, suggesting that proper folding of these smaller protein constructs was largely maintained on the ribosome. Constructs containing either LRRs 6–9 or LRRs 21–24 from LeEIX2, which had high signal-to-noise ratios, were used to show that signal in an EIX competition assay is dose-dependent (Fig. 4B, Supplementary Fig. 8). An additional binding assay in RD format demonstrated that sub-segment binding is specific to EIX by comparing to non-specific interactions with casein as an immobilized target or as a competitor in solution (Fig. 4C, Supplementary Fig. 8). Collectively, these results provide a body of evidence for two disjoint EIX binding hotspots on LeEIX2.

### Analogous binding hotspots are found in the decoy receptor LeEIX1

**Figure 5:**
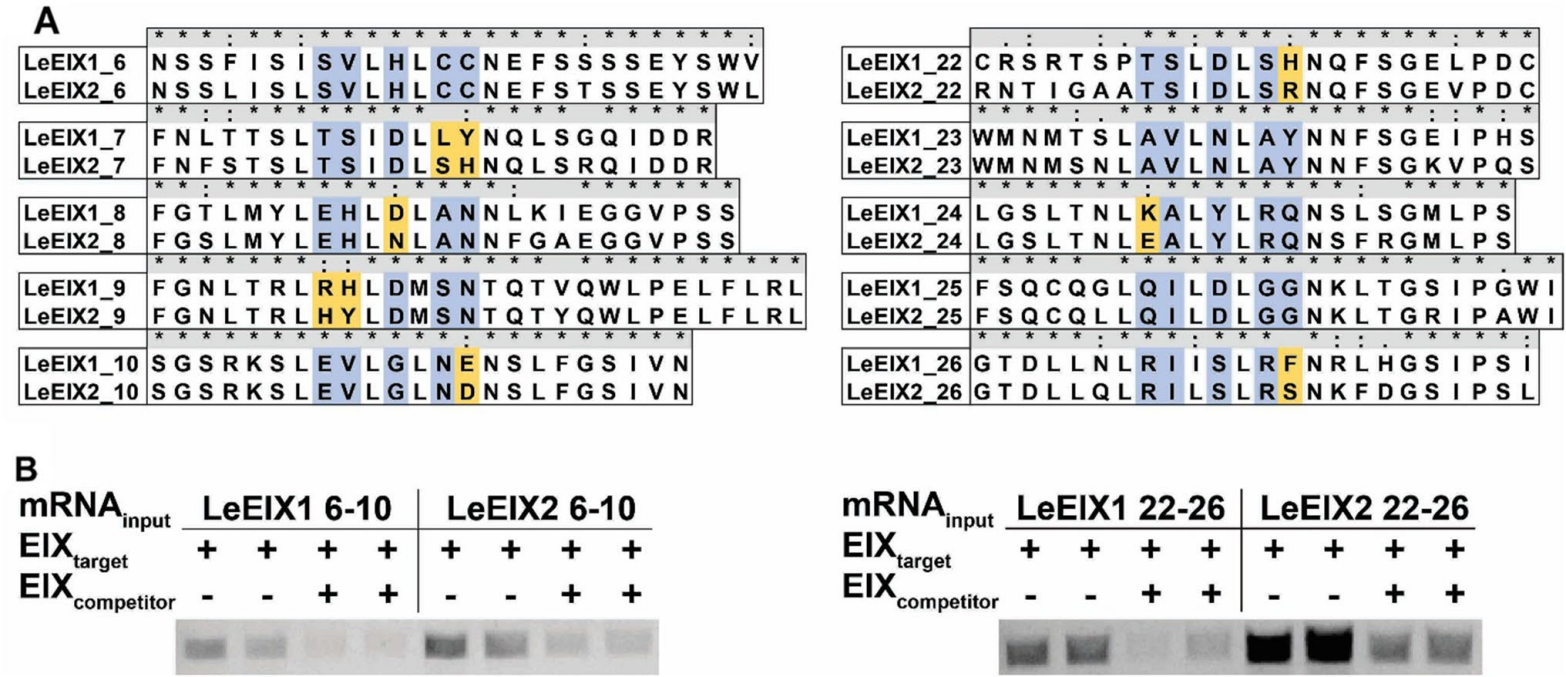
Hotspot comparison between LeEIX1 and LeEIX2. (**A**) Amino acid sequence comparison between LeEIX1 and LeEIX2 for sub-segment LRRs 6–10 (left) and 22–26 (right). Residue comparison is indicated as fully conserved (*), strongly similar (:), or weakly similar (.), with emphasis on target-facing residues through coloring as fully conserved (blue) or not fully conserved (orange). (**B**) RD results of LeEIX1 and LeEIX2 sub-segments LRRs 6–10 (left) or 22–26 (right) panning against immobilized EIX in the absence or presence of excess competitor EIX.

LeEIX1 is posited to be a decoy receptor in the EIX response network; this receptor is known to bind EIX but cannot signal a hypersensitive response upon target recognition^17^. The unexpected result of a disjoint paratope in LeEIX2 led us to test a new hypothesis that LeEIX1 may lack one of the two hotspots for binding to EIX, leading to the observed phenotype. We first compared the amino acid sequences of both receptors at the identified LeEIX2 hotspot LRRs (Fig. 5A). Although there were differences at this level, even at the likely target-facing residues based on similar LRR receptor interactions, it remained unclear what role these mutations may have. We therefore performed analytical RD using the analogous LRRs 6–10 and 22–26 of LeEIX1 (Fig. 5B, Supplementary Fig. 9). The results indicate that both LeEIX1 hotspot sites appear to be functional in binding EIX.

## Discussion

Plant LRR receptors have long remained a recalcitrant class for heterologous protein expression in *E. coli*, so here we used a cell-free method, RD, to dissect and identify subsets of LRRs implicated in effector binding. RD has previously been used to analyze protein–protein interactions, but the signal-to-noise ratio is dependent on the size of the protein of interest and previous reports indicate poor efficiency in displaying proteins larger than ~ 70 kDa^14^. Given the notable size of the LeEIX2 ectodomain (~ 115 kDa), its successful display in RD was achieved by using a fully defined cell-free translation system, codon optimization, more efficient blocking of non-specific interactions, and in silico-informed optimization of the reverse transcription primer (Supplementary Fig. 4A,B). To our knowledge, the LeEIX2 ectodomain is the largest protein reported to be functionally displayed on the ribosome. Since ribosome display excludes the natural receptor milieu, its utility in decoupling receptor binding from signaling is appropriate for the study of direct interactions, as is the case in this study.

We also utilized an information entropy approach to identify optimal sub-segmentation sizes and to facilitate identification of more focused binding hotspots in both LeEIX2 and LeEIX1. Given that many plant immune receptors are as similarly large as LeEIX2 and can also be sub-segmented due to the modular LRR sequences in their ectodomains, these methodological improvements in RD now pave the way for significant advancements in elucidating the molecular basis for plant immune recognition. The cell-free context must also be considered, as the environment may require adjustments for protein–protein interactions that necessitate co-receptors or other environmental factors to augment the interaction affinity. The adaptability of the cell-free approach readily accommodates such adjustments.

The finding of two discontiguous LeEIX2 binding hotspots suggests that either EIX is bound at two distinct paratopes, contrasting the single epitope that has been the exclusive focus in literature^18–20^, or LeEIX2 can bind two EIX molecules. A hypothetical structural model for a putative one-to-one interaction via two paratopes suggests that this is physically possible (Supplementary Fig. 10), although a two-to-one interaction may occur if LeEIX2 adopts a more extended conformation. This unexpected result for LeEIX2 expands our understanding of how LRR receptors may recognize their cognate targets and control the signaling response to this recognition.

As both LeEIX1 and LeEIX2 share analogous EIX-binding domains, the distinct phenotypic responses from the two receptors likely arise in downstream signaling, highlighting an advantage of cell-free RD over in vivo approaches by decoupling binding and signaling. Another interesting implication of this finding is that it provides a potential mechanism for EIX to act as a molecular glue holding the two receptors together as a heterodimer, an observation witnessed previously in literature^17^. Bivalent ligand-induced dimerization has been seen before for pattern recognition immune receptor AtCERK1 binding via chitin oligomers^21^, so there is some precedent for such a mechanism.

Our optimized RD method should enable elucidation of plant immune receptor binding determinants, even when the protein itself cannot be heterologously expressed. Although demonstrated here with tomato receptors that recognize EIX, the approach should be applicable to any modular plant immune receptor with a known ligand. It can also be used to discover new interactions by screening a displayed receptor against a host of possible targets or by employing a library of immune receptors in RD and sequencing the DNA product recovered after binding.

## Materials and methods

### Target proteins

EIX was purchased as purified protein (Megazyme or Sigma-Aldrich) and reconstituted at 167 µM in 1× TBS, pH 7.6. MBP was produced in BL21(DE3) *Escherichia coli* and purified using Ni–NTA affinity chromatography. Target purity was confirmed via SDS-PAGE (Supplementary Fig. 11). Casein was purchased (Sigma-Aldrich) and reconstituted at 167 µM total protein in 1× TBS, pH 7.6 assuming an average molecular weight of 22.5 kDa for the heterogeneous mixture.

### DNA cloning

The amino acid sequence for the LeEIX2 ectodomain was codon-optimized for *E. coli* using the browser-based codon optimization tool from Integrated DNA Technologies (IDT), as subsequent experimentation involved expression in the *E. coli*-derived PURExpress translation system (New England Biolabs). The corresponding DNA was ordered from IDT as two separate gBlocks. Each gBlock was resuspended in water to 100 nM, PCR amplified, and then digested using BsaI-HF restriction enzyme. The two pieces were ligated and gel extracted. The ligated product was digested using BbsI and then column purified to isolate ligated product with sticky ends. The full LeEIX2-encoding DNA was cloned into BbsI-digested pRDV2 plasmid^22^, containing a 5′ T7 promoter/ribosome binding site (RBS) and a 3′ tolA spacer sequence (which fills the ribosomal tunnel to allow the full displayed protein to exit and fold). The ligation product was transformed into *E. coli*, and the purified plasmid product was verified by Sanger sequencing.

LeEIX2 segments of interest were PCR amplified from pRDV2-LeEIX2 template and digested with SapI. A three-piece linear ligation was performed with the T7 promoter + N-cap (piece 1), digested LeEIX2 sub-segment (piece 2), and C-cap + tolA (piece 3). LeEIX1 segment gBlocks were digested with SapI and ligated in an analogous three-piece linear ligation. Pieces and their ligation products were verified on an agarose gel. This linear DNA served as the template for segment analysis.

### mRNA synthesis

pRDV2 containing LeEIX2 ectodomain or the desired segment was PCR amplified in a 50 µL reaction for 30 cycles with Q5 polymerase using primers T7B_noBsaI and tolAk. These primers encode for a 5′ T7 promoter and RBS, and a 3′ tolA sequence. Notably, the reverse primer does not encode for a stop codon after the gene to prevent ribosome release during the later translation step. Correct PCR product was verified on an agarose gel. The PCR product was used for in vitro transcription using T7 RNA polymerase. The resulting mRNA was then purified through a series of ethanol-salt precipitation steps and quantified via spectrophotometry in a Cytation3 Imaging Reader (BioTek)^23^.

### In situ ribosome display

For EIX-immobilized wells, 100 µL of 66.7 nM EIX in 1× TBS, pH 7.6 solution were added to the bottom of each well of a Maxisorp strip (Nunc). For unrelated target wells, 100 µL of 66.7 nM MBP were added. The wells were kept at 4 °C overnight. The solutions were removed, and 100 µL SynBlock buffer (Bio-Rad) were added as a blocking agent for at least three hours at 4 °C and subsequently removed.

Separately, translation mixes were prepared using the PURExpress system with 5.0 × 10^12^ mRNA molecules as template and 4.5 × 10^13^ ribosomes per tube of 13 µL solutions. Reactions were run for 30 min at 37 °C and stopped by adding 90 µL ice cold WBT (1× TBS, pH 7.6, 50 mM MgCl_2_, 0.05% Tween-20) per tube to generate the stable mRNA-ribosome-protein complexes. The lack of a stop codon in the transcript causes the ribosome to stall during the translation process, creating the RD complexes^24^. After translation, 0.75 µL of 167 µM competitor EIX protein, corresponding to 6.8 × 10^13^ molecules, were added to the appropriate competition tubes to a final concentration of 1.25 µM. In each well, 100 µL of the translation mix were added and panning was performed at 4 °C for 1 h.

The wells were then washed with 300 µL WBT in progressively more stringent washes of 3 × instant, 3 × 12 s, and 3 × 30 s. The complexes were incubated at 70 °C for 10 min in the presence of 16.25 µL of 1.92 µM RD_mid_rev primer (for PCR) or tolAk primer (for qPCR). Then, in situ reverse transcription was performed with AffinityScript (Agilent) at 42 °C for 1 h.

The resulting cDNA (0.5 µL) was the template for PCR using RD_in_for and pRDV_BbsI_r primers, and this product was visualized on an agarose gel to compare complexes bound in each RD well. Relative signal intensities were quantified using the Image Lab 6.1 (Bio-Rad) band quantification tool. Alternatively, the cDNA (1 µL) was the template for qPCR using qPCR1.F and RD_mid_rev primers. Analysis of the qPCR results was performed using LightCycler 96 1.1 (Roche) and Excel (Microsoft).

### Reverse transcription primer optimization

Initial RD attempts at reproducing the LeEIX2-EIX interaction were unsuccessful. After troubleshooting, it became clear that the reverse transcription step was the cause of failure. The LeEIX2-coding RNA sequence was input to Mfold^16^, and it was discovered that the complementary RNA strand for the standard reverse transcription primer pRDV_BbsI_r may form secondary structure with a LeEIX2 RNA segment at reverse transcription temperatures, preventing primer annealing (Supplementary Fig. 4A). Accordingly, reverse transcription primer RD_mid_rev was designed to anneal to a region of lower secondary RNA structure (Supplementary Fig. 4B).

### Sequence comparison

Sequences for LeEIX1 and LeEIX2 were compared using ClustalX software^25^. Expected paratope residues were determined using a combination of LRR consensus design, structural information for LRRs in plants and other organisms, and a mutual information analysis of *Solanaceae* LRRs.

### Statistical analysis

The qRT-PCR Cq data from the independent replicates of the 5-LRR sub-segment RD experiments were normalized to the same global average Cq to enable robust calculation of means and standard deviations from samples across different days. To compare binding specificities, ΔCq values were calculated with standard deviations of these differences estimated through error propagation of the component Cq standard deviations. A single sample (LRRs 23–27) with an outlying standard deviation was excluded from the statistical analysis following Tukey’s heuristic of outliers (Supplementary Fig. 12). Single-factor ANOVA was performed on the ΔCq distributions, with Tukey’s test performed to determine which 5-LRR sub-segments showed statistically greater signal than others tested.

## Supplementary Information

Supplementary Information 1.

Supplementary Information 2.
