# Supplementary material for: A cell-free approach to identify binding hotspots in plant immune receptors
Source: Sci Rep. 2022 Jan 11;12:501. doi: 10.1038/s41598-021-04259-8 (PMC8752824; doi:10.1038/s41598-021-04259-8)
Supplement: Supplementary file 1 — Supplementary Information 1. [file 41598_2021_4259_MOESM1_ESM.pdf]

Supplementary Information for:

A Cell-Free Approach to Identify Binding Hotspots in Plant Immune Receptors

George C. Markou[1] and Casim A. Sarkar[2,*]

[1] Department of Chemical Engineering and Materials Science, University of Minnesota, Minneapolis, MN 55455, USA

[2] Department of Biomedical Engineering, University of Minnesota, Minneapolis, MN 55455, USA

[*] Corresponding author: C. A. Sarkar; E-mail: csarkar@umn.edu

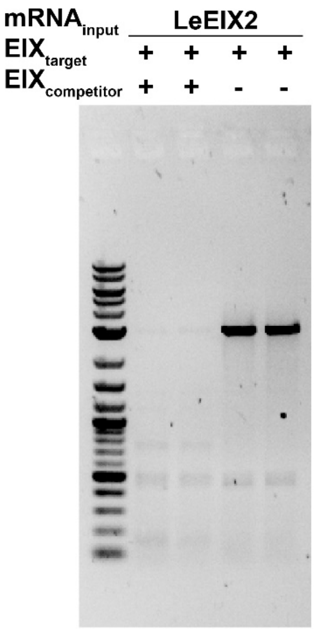

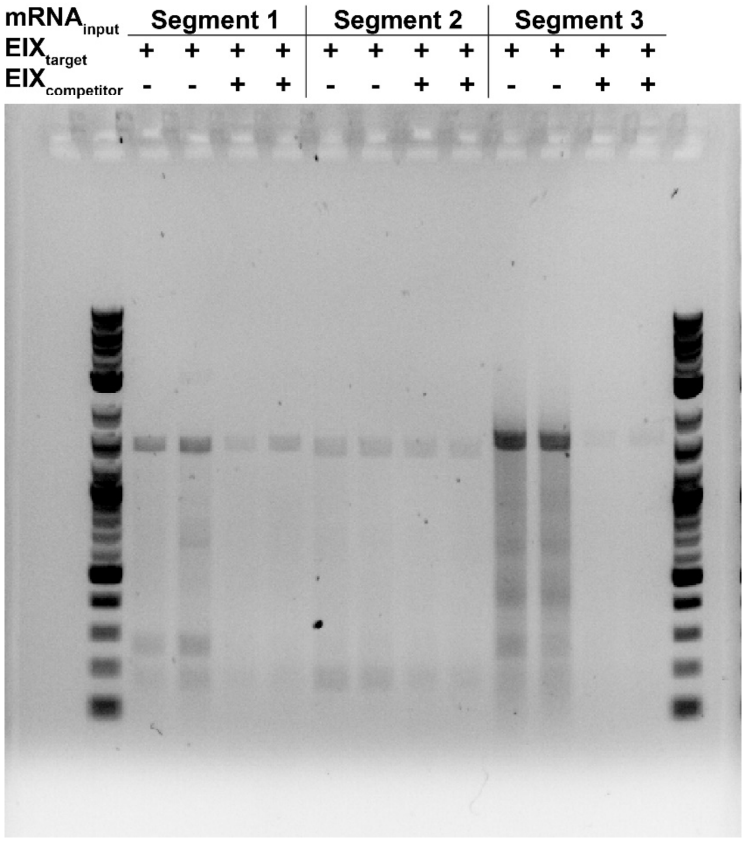

Supplementary Figure 1. Unprocessed gel images for Figure 1. Reference ladder is the 1 kb Plus DNA Ladder (New England Biolabs).

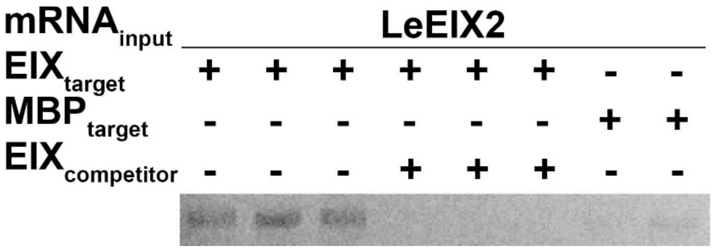

Supplementary Figure 2. Ribosome display of LeEIX2 panned against immobilized EIX target in the absence or presence of excess EIX competitor performed in triplicate. Ribosome display of LeEIX2 panned against an unrelated immobilized protein, maltose-binding protein, was also performed in duplicate. As noted in the main text, significant modifications to the ribosome display protocol were required to obtain this signal clarity for the full LeEIX2 ectodomain.

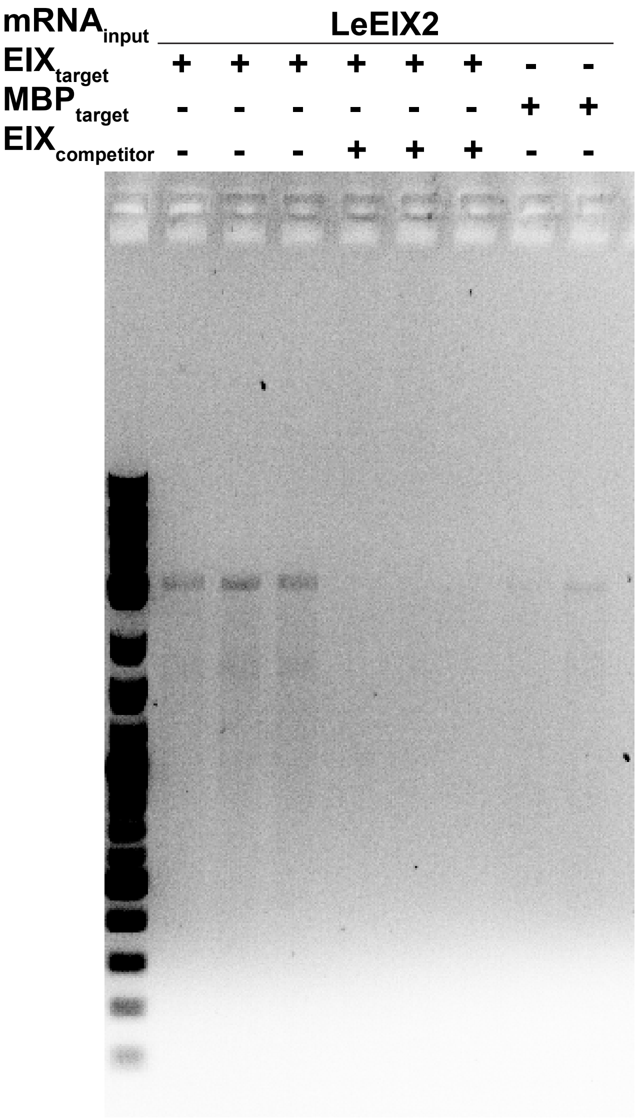

Supplementary Figure 3. Unprocessed gel image for Supplementary Figure 2. Reference ladder is the 1 kb Plus DNA Ladder (New England Biolabs).

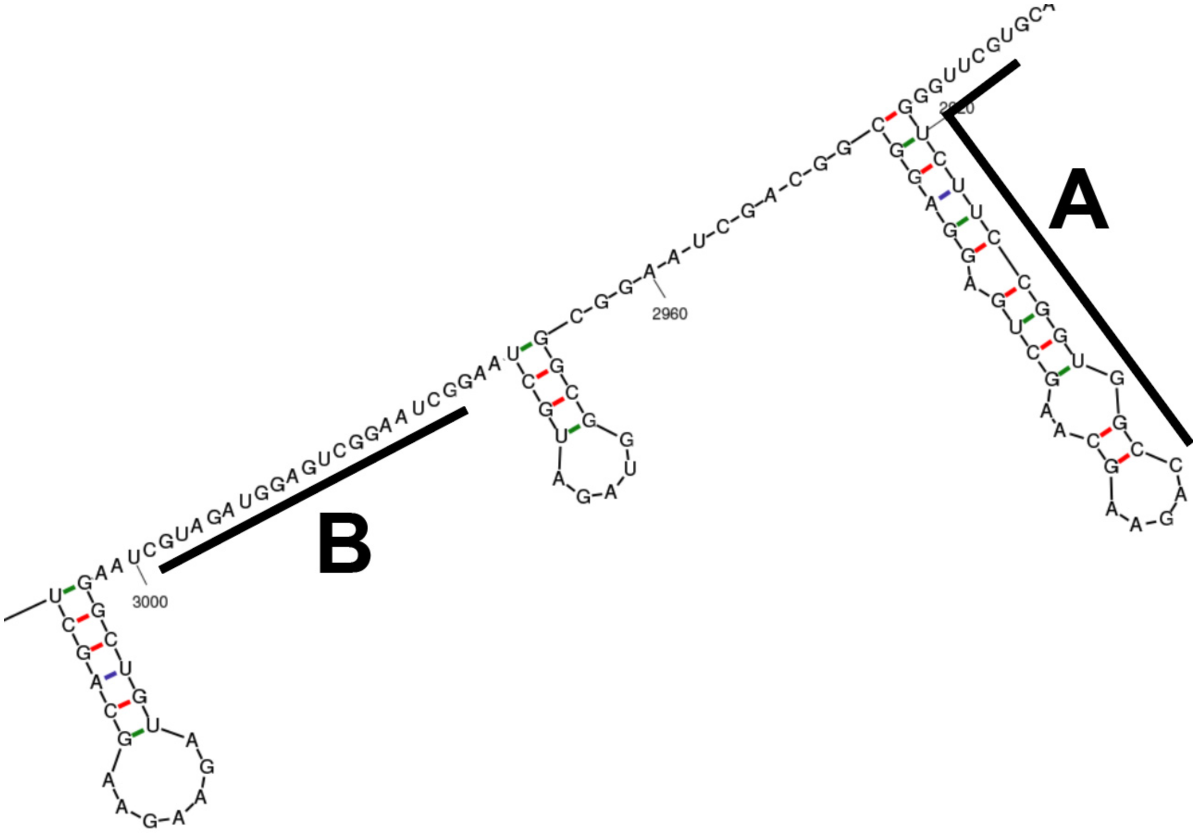

Supplementary Figure 4. Mfold RNA folding results for LeEIX2 transcript. Black bars indicate reverse transcription (RT) annealing site of **(A)** primer pRDV_BbsI_r or **(B)** primer RD_mid_rev. RT-PCR using the standard ribosome display RT primer pRDV_BbsI_r failed, likely due to the RNA secondary structure at these temperatures. Reverse transcription primer RD_mid_rev proved successful, owing to the more unstructured RNA sequence to which it anneals.

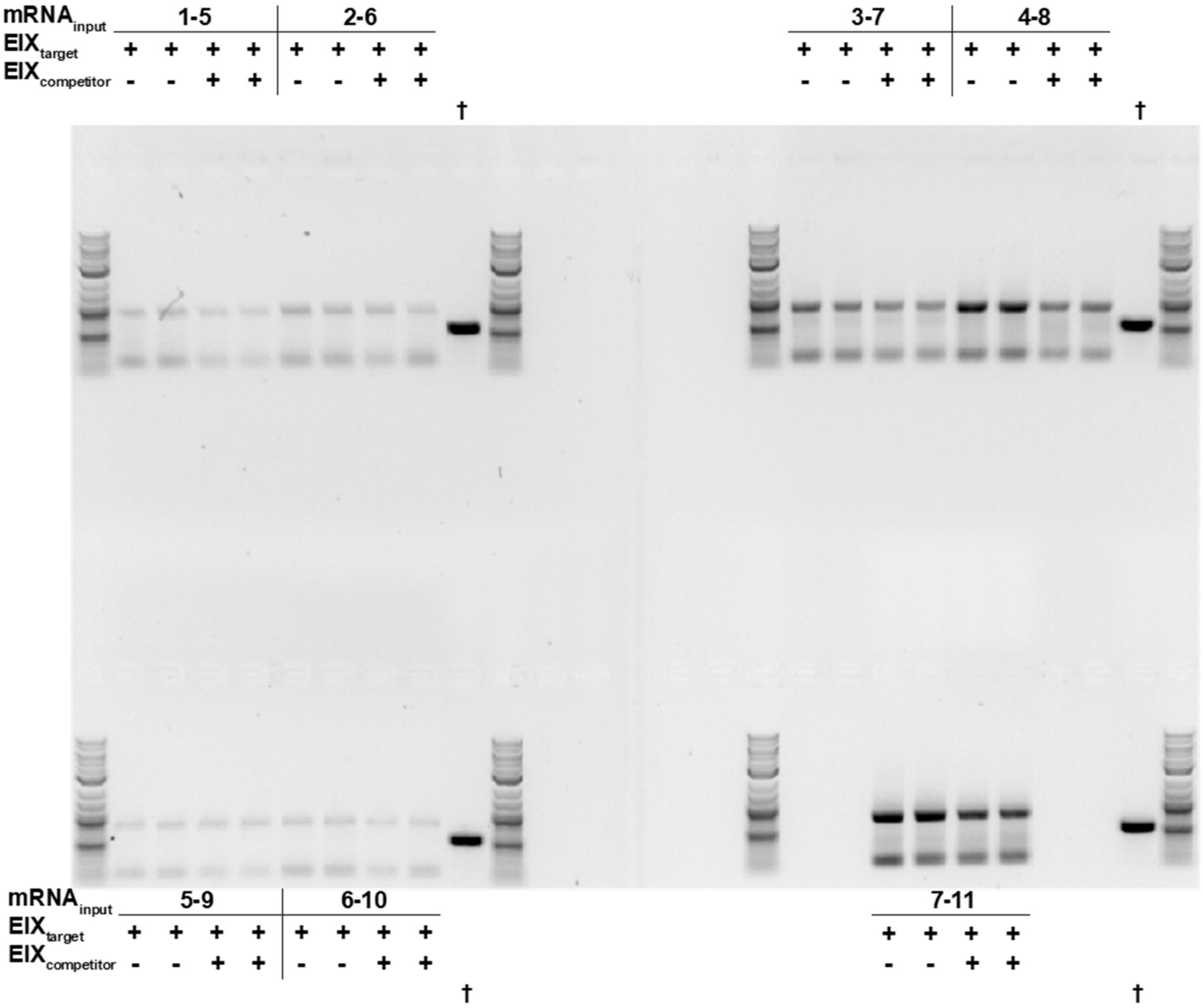

Supplementary Figure 5. Unprocessed gel image for Figure 3A. Reference ladder is the 1 kb Plus DNA Ladder (New England Biolabs).

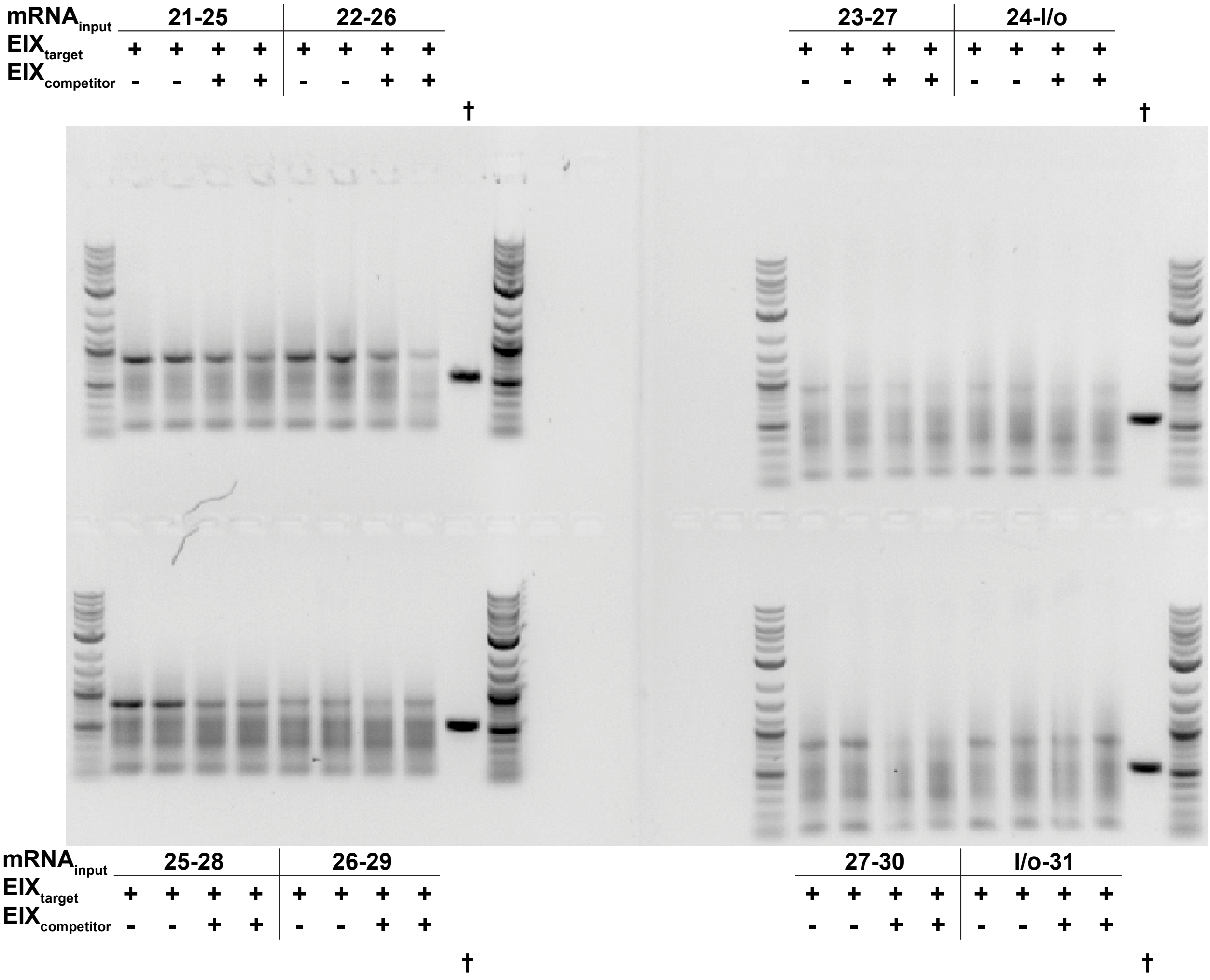

Supplementary Figure 6. Unprocessed gel image for Figure 3B. Reference ladder is the 1 kb Plus DNA Ladder (New England Biolabs).

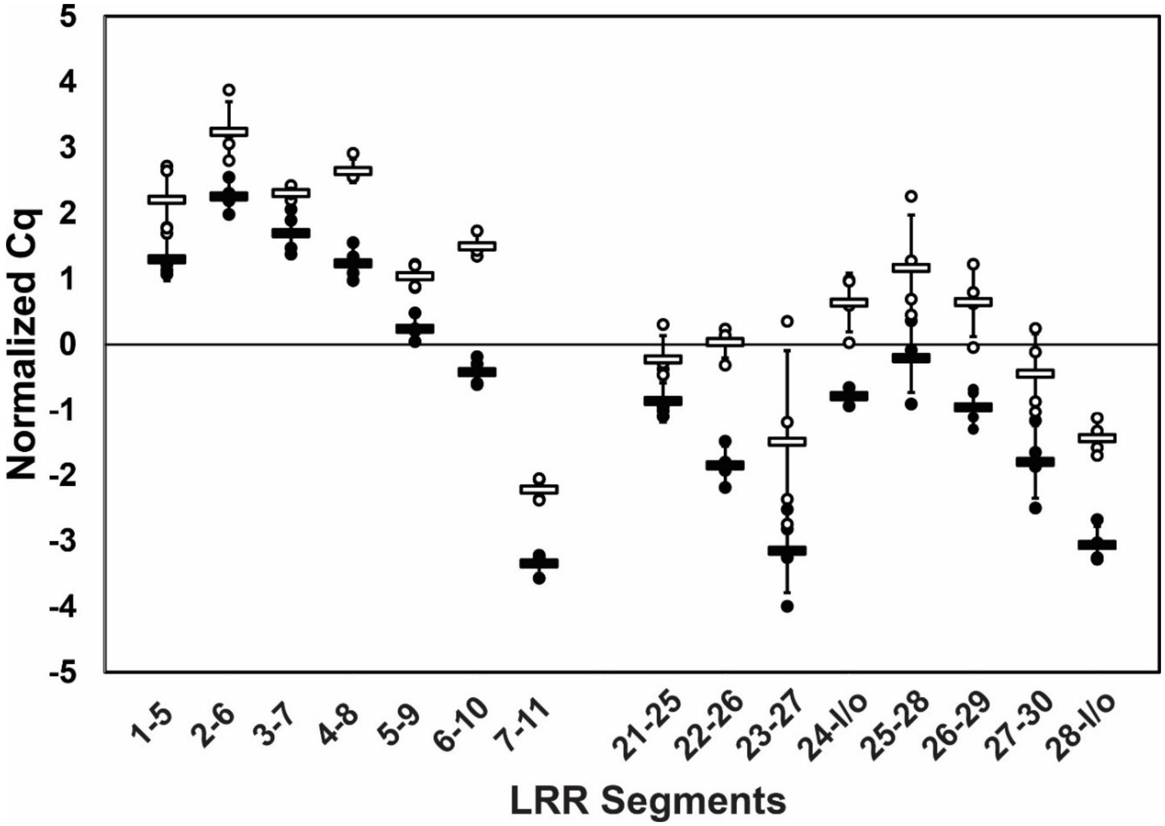

Supplementary Figure 7. Cq values measured using qRT-PCR for 5-LRR sub-segment RD experiments of wells without (white circle) or with (black circle) excess competitor EIX. Data are normalized such that the average global Cq from two sets of different experimental days was set to 0. Means (white or black rectangle) and standard deviations (error bars) for each set of replicates are shown.

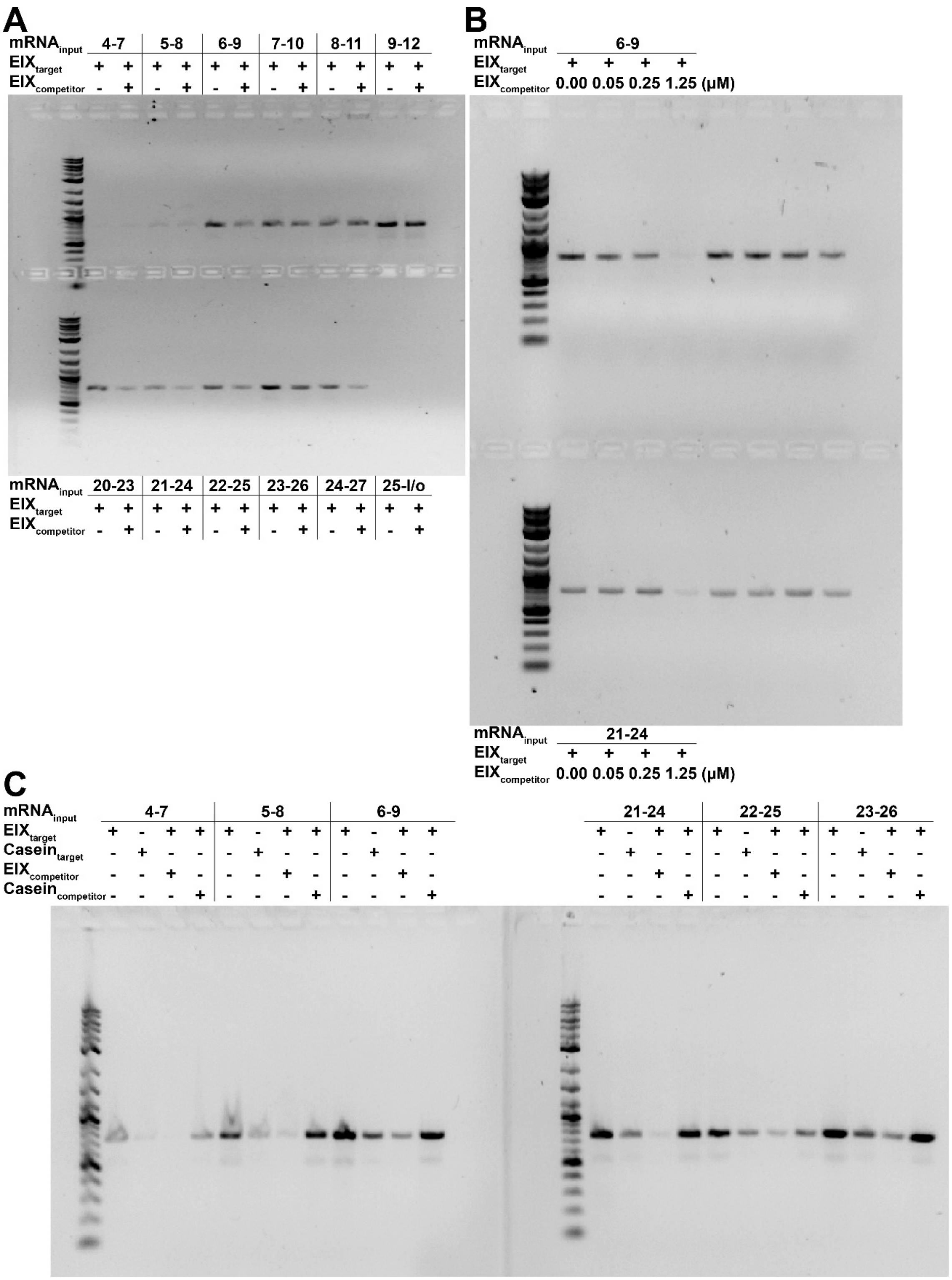

Supplementary Figure 8. Unprocessed gel images for Figure 4. Reference ladder is the 1 kb Plus DNA Ladder (New England Biolabs). The rightmost four sample lanes in the top and bottom halves of (B) are from an unrelated experiment.

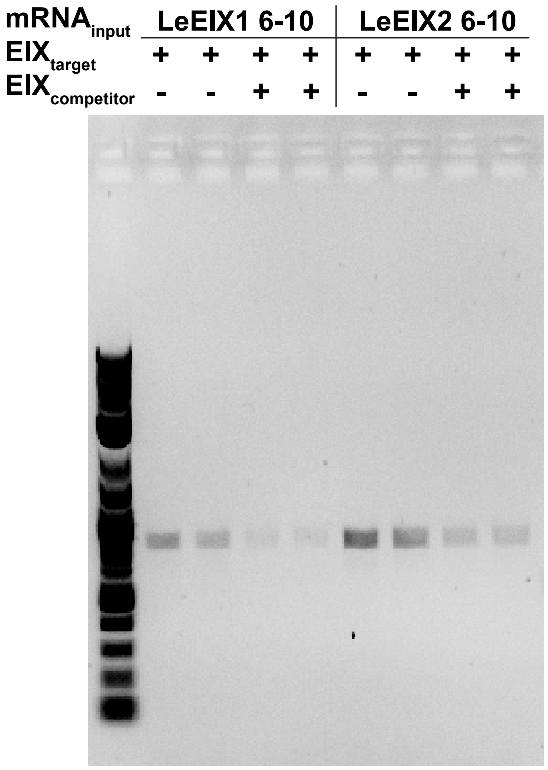

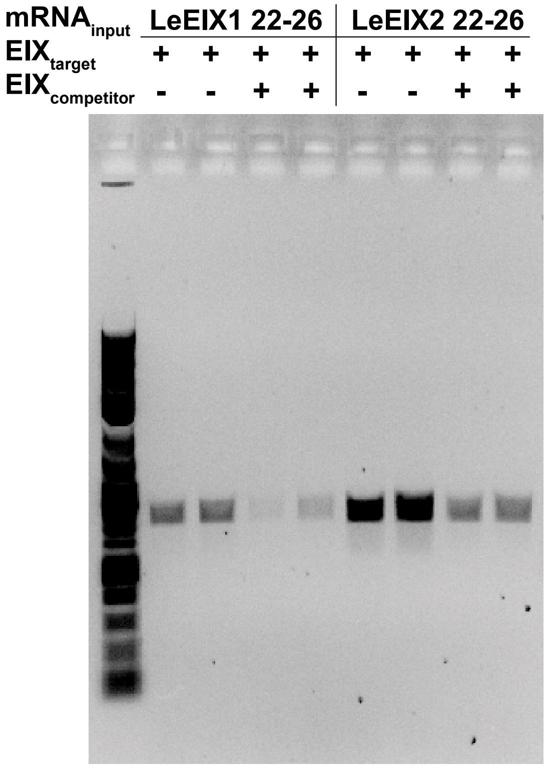

Supplementary Figure 9. Unprocessed gel images for Figure 5B. Reference ladder is the 1 kb Plus DNA Ladder (New England Biolabs).

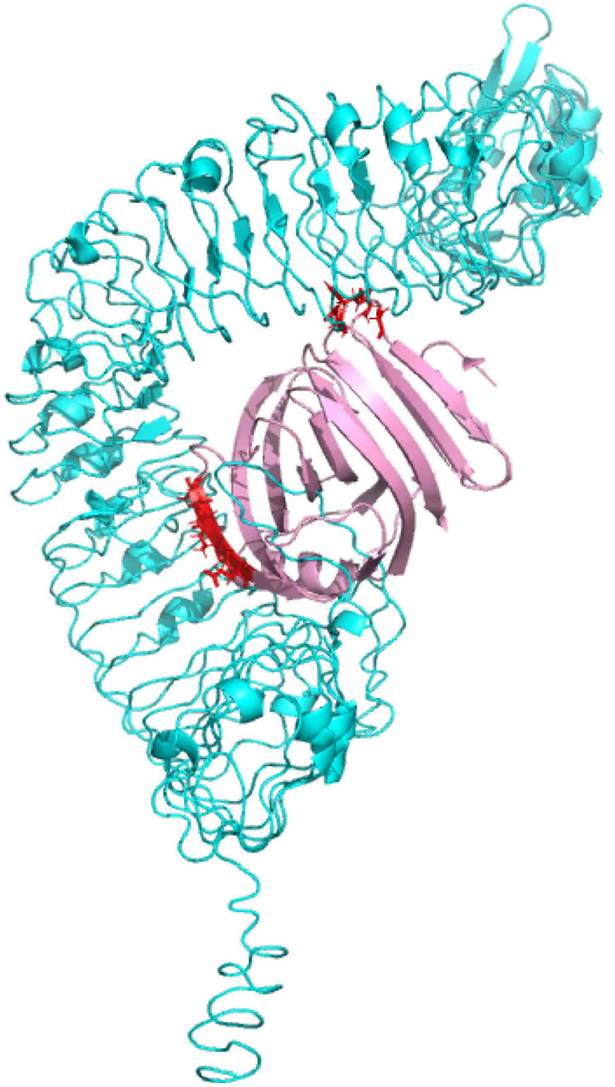

Supplementary Figure 10. Hypothetical model of LeEIX2 (cyan) interacting with EIX (pink) in a 1:1 stoichiometry. As a proxy for the 3D structure of EIX, the crystal structure for Xylanase II from *Trichoderma reesei* (3LGR)[1] was imported from RCSB PDB into PyMOL. Since neither LeEIX2 nor any homologs have resolved crystal structures, the 3D structure of LeEIX2 was estimated by submitting its amino acid sequence to I-TASSER[2] and importing the output into PyMOL. The purported EIX epitope TKLGE is shown in red here interacting with LRRs 22-26 and a disjoint epitope on EIX is shown in red in the proximity of LRRs 6-10. This hypothetical LeEIX2 structural model only serves to highlight the physical possibility for two hotspots to accommodate a single EIX molecule, although a more extended conformational state may favor two EIX molecules interacting with the two distinct paratopes in LeEIX2.

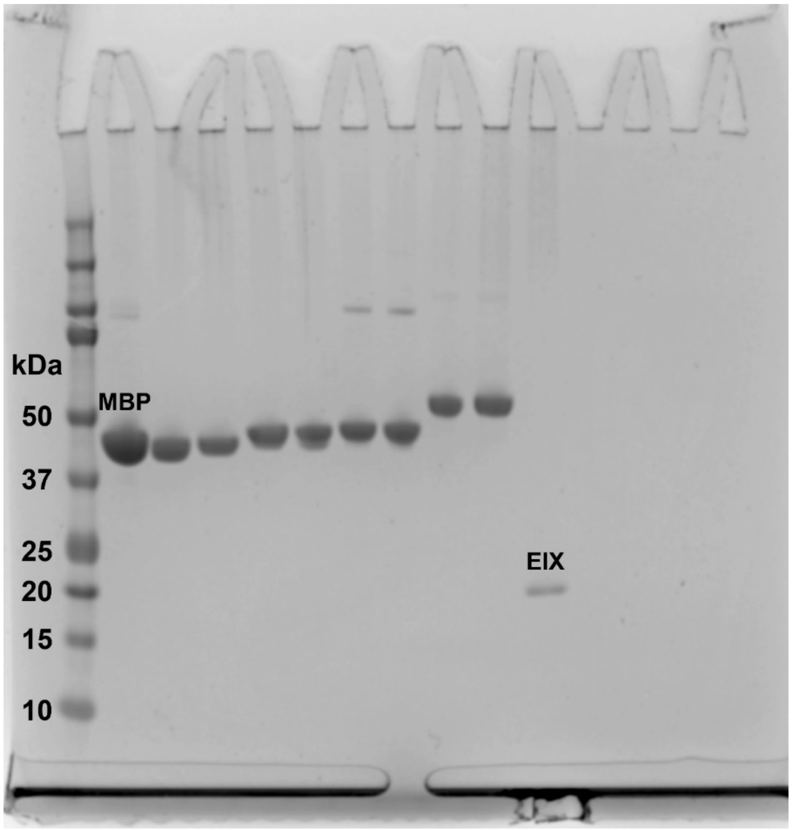

Supplementary Figure 11. SDS-PAGE image of the two pure proteins used in this study, maltose-binding protein (MBP; ~43 kDa) and ethylene-inducing xylanase (EIX; ~21 kDa). Reference ladder is the Precision Plus Protein Kaleidoscope Prestained Protein Standards (Bio-Rad). All unlabeled lanes are from an unrelated experiment.

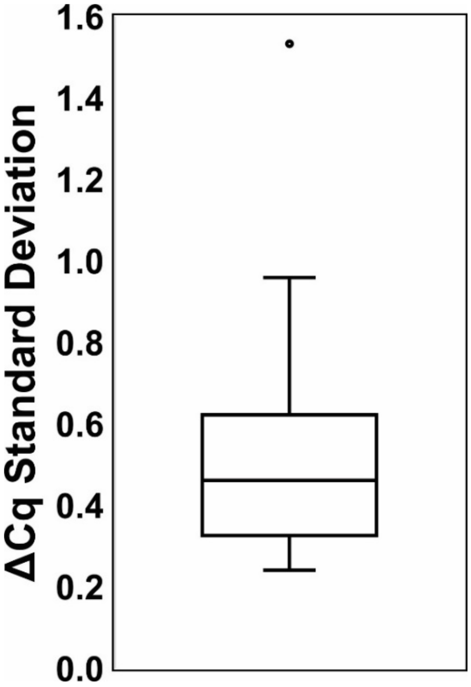

Supplementary Figure 12. Box plot of the ΔCq standard deviations to determine if there were any outliers in the measured data. Following Tukey's heuristic of outliers lying outside the limit of 1st quartile - 1.5×(interquartile range) or 3rd quartile + 1.5×(interquartile range), the error-propagated standard deviation for the calculated ΔCq of LRRs 23-27 (data point shown) was substantially outside the range and was therefore excluded in the statistical analysis to determine binding hotspots.

LITERATURE CITED

1. Pompidor, G.; Maury, O.; Vicat, J.; and Kahn, R. A dipicolinate lanthanide complex for solving protein structures using anomalous diffraction. *Acta Crystallographica* **D66,** 762-769 (2010).

2. Yang, J.; Yan, R.; Roy, A.; Xy, D.; Poisson, J.; and Zhang, Y. The I-TASSER Suite: Protein structure and function prediction. *Nature Methods* **12,** 7-8 (2015).
